# Factors Influencing Clinician Trust in Predictive Clinical Decision Support Systems for In-Hospital Deterioration: Qualitative Descriptive Study

**DOI:** 10.2196/33960

**Published:** 2022-05-12

**Authors:** Jessica M Schwartz, Maureen George, Sarah Collins Rossetti, Patricia C Dykes, Simon R Minshall, Eugene Lucas, Kenrick D Cato

**Affiliations:** 1 Department of Biomedical Informatics Columbia University New York, NY United States; 2 School of Nursing Columbia University New York, NY United States; 3 Brigham and Women's Hospital Boston, MA United States; 4 Harvard Medical School Boston, MA United States; 5 School of Health Information Science University of Victoria Victoria, BC Canada; 6 Weill Cornell Medicine New York, NY United States; 7 Department of Emergency Medicine Columbia University New York, NY United States

**Keywords:** clinical decision support systems, machine learning, inpatient, nurses, physicians, qualitative research

## Abstract

**Background:**

Clinician trust in machine learning–based clinical decision support systems (CDSSs) for predicting in-hospital deterioration (a type of predictive CDSS) is essential for adoption. Evidence shows that clinician trust in predictive CDSSs is influenced by perceived understandability and perceived accuracy.

**Objective:**

The aim of this study was to explore the phenomenon of clinician trust in predictive CDSSs for in-hospital deterioration by confirming and characterizing factors known to influence trust (understandability and accuracy), uncovering and describing other influencing factors, and comparing nurses’ and prescribing providers’ trust in predictive CDSSs.

**Methods:**

We followed a qualitative descriptive methodology conducting directed deductive and inductive content analysis of interview data. Directed deductive analyses were guided by the human-computer trust conceptual framework. Semistructured interviews were conducted with nurses and prescribing providers (physicians, physician assistants, or nurse practitioners) working with a predictive CDSS at 2 hospitals in Mass General Brigham.

**Results:**

A total of 17 clinicians were interviewed. Concepts from the human-computer trust conceptual framework—*perceived understandability* and *perceived technical competence* (ie, perceived accuracy)—were found to influence clinician trust in predictive CDSSs for in-hospital deterioration. The concordance between clinicians’ impressions of patients’ clinical status and system predictions influenced clinicians’ perceptions of system accuracy. Understandability was influenced by system explanations, both global and local, as well as training. In total, 3 additional themes emerged from the inductive analysis. The first, *perceived actionability*, captured the variation in clinicians’ desires for predictive CDSSs to recommend a discrete action. The second, *evidence,* described the importance of both macro- (scientific) and micro- (anecdotal) evidence for fostering trust. The final theme, *equitability*, described fairness in system predictions. The findings were largely similar between nurses and prescribing providers.

**Conclusions:**

Although there is a perceived trade-off between machine learning–based CDSS accuracy and understandability, our findings confirm that both are important for fostering clinician trust in predictive CDSSs for in-hospital deterioration. We found that reliance on the predictive CDSS in the clinical workflow may influence clinicians’ requirements for trust. Future research should explore the impact of reliance, the optimal explanation design for enhancing understandability, and the role of perceived actionability in driving trust.

## Introduction

### Background

Clinician adoption of clinical decision support systems (CDSSs) is crucial if best practices are to be integrated into standard clinical workflows. With CDSSs evolving to include machine learning–based CDSSs, the power of machine learning can be leveraged to enhance predictive models of patient risk for a diagnosis or outcome. However, such systems face unique challenges to adoption compared with systems using rule-based logic, which have historically been more widely implemented [1]. A challenge is that the logic behind predictions in machine learning–based CDSSs can be difficult or impossible to make transparent to clinical end users. This has been the focus of much recent research [2-4] in response to the European Union General Data Protection Regulation that effectively mandates a right to explanation of any prediction made based on a person’s data [5]. In a study of physicians’ ability to understand and explain a machine learning–based CDSS’s logic, Diprose et al [6] found that both understandability and explainability were positively associated with *trust*. When the logic behind the predictions was not understood, the physicians did not trust them. Such distrust has been shown to challenge the adoption of machine learning–based CDSSs [7-10], whereas trust is associated with increased intent to adopt machine learning–based CDSSs [11].

Machine learning–based early warning systems, a popular type of CDSS [12], aim to identify patients at risk of deteriorating in the hospital (eg, developing sepsis or experiencing cardiac arrest). These are a type of *predictive CDSS***—**machine learning–based systems that make predictions about future patient outcomes or responses to treatment. Predictive CDSSs present more difficulty for clinicians to trust compared with machine learning–based *diagnostic* CDSSs as they require the clinician to trust the accuracy of the prediction even in the absence of objective signs of the outcome. It has been difficult for predictive CDSSs to achieve impactful adoption [10,13,14]. Research indicates that presenting clinicians with a model’s overall accuracy is not sufficient for establishing trust in predictive CDSSs [2]. Therefore, how clinicians come to trust and adopt predictive CDSSs remains an area of intense research interest. Moreover, most research on clinician trust has focused on physicians’ trust in predictive CDSSs and machine learning–based diagnostic CDSSs [2,6,15]. However, nurses are also target users of predictive CDSSs in the hospital setting [7,16] and may have different perceptions of and requirements for trusting predictive CDSSs.

Others have investigated this topic. For example, a study aimed to explore the factors that influence the integration of predictive CDSSs into clinical workflows and found trust to be an influencing factor in the emergency department [7]. Others have explored the factors that influence explainability and characterize when explainability increases trust [2]. Another study tested physicians’ trust in predictive CDSSs given exposure to different explanations and levels of understanding [6]. The latter two were conducted by referring to simulated as opposed to live implemented predictive CDSSs. Our study is unique from existing research on this topic as it is the first with the objective of elucidating the factors that influence trust referring to an implemented, in-use system in a broad inpatient hospital setting (medical, surgical, and intensive care units).

### Objectives

To address this gap in our understanding of how clinicians establish trust in predictive CDSSs and how this might differ by professional group, we explore the experiences of nurses and prescribing providers (physicians, physician assistants [PAs], or nurse practitioners) after the implementation of a predictive CDSS for in-hospital deterioration. Our investigation is guided by a conceptual framework, the human-computer trust framework [17], which accounts for the aforementioned known factors that influence trust—perceived understandability and accuracy. Thus, the objective of our study was to explore the phenomenon of clinician trust among users of a predictive CDSS for in-hospital deterioration by (1) confirming and characterizing the human-computer trust concepts, (2) uncovering and describing any other factors that influence clinician trust, and (3) comparing nurses’ and prescribing providers’ trust in predictive CDSSs.

## Methods

### Conceptual Framework

**Figure 1:**
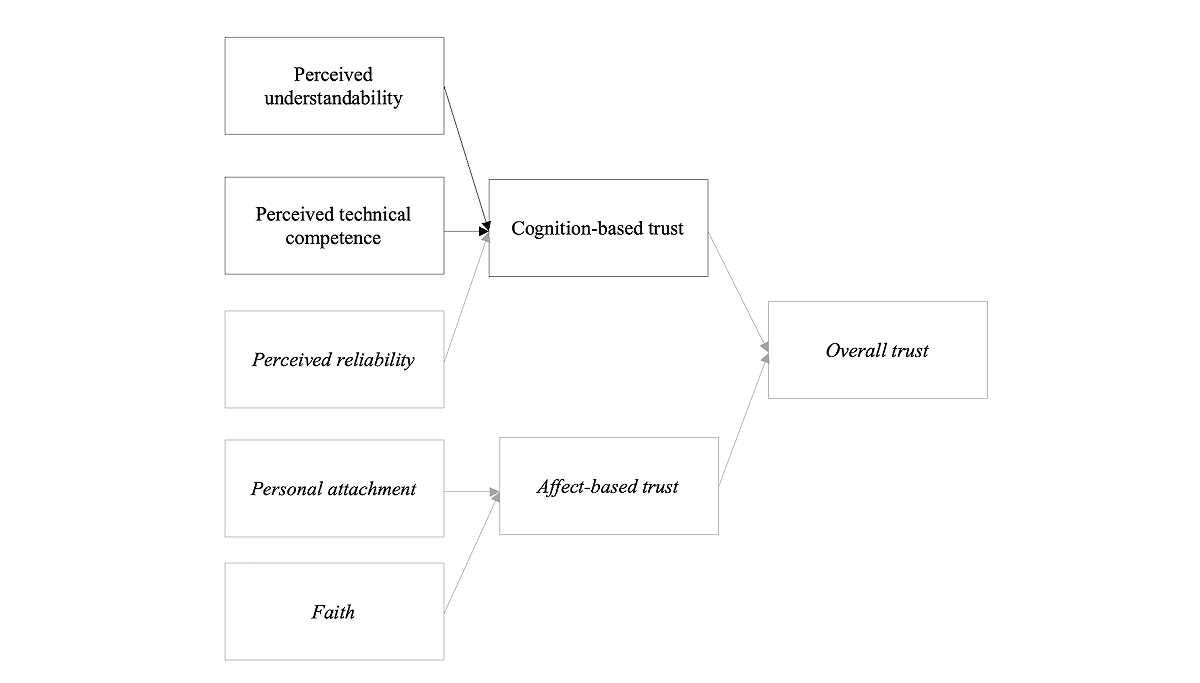
Human-computer trust conceptual framework. Perceived understandability, perceived technical competence, and cognition-based trust were investigated in this study.

The *human*-*computer trust* conceptual framework [17] (Figure 1) guided our investigation. In the framework, overall trust is defined as “the extent to which a user is confident in, and willing to act on the basis of, the recommendations, actions, and decisions of an artificially intelligent decision aid” [17]. In this framework, trust is further characterized as cognition-based trust (reflective of the user’s intellectual perceptions of the system) and affect-based trust (reflective of the user’s emotional perceptions of the system). This study focused on the experience of cognition-based trust and 2 of its concepts: perceived understandability and perceived technical competence. Perceived understandability is defined as “the sense that the human supervisor or observer can form a mental model and predict future system behavior” [17]. Perceived technical competence is defined as “the system is perceived to perform tasks accurately and correctly based on the information that is input” [17]. Although perceived understandability and perceived technical competence are related concepts—ideally, a clinician will understand a system to judge its accuracy—the inclusion of *perceived* with each concept puts the emphasis on clinicians’ perspectives and how those perspectives influence trust whether they are accurate or not.

We did not directly examine perceived reliability because it depends on repeated system use, which was not guaranteed among the participants, and because structural equation modeling led investigators to question its influence on cognition-based trust [17]. Thus, our line of inquiry was focused on participants’ perceptions of their understanding of a predictive CDSS, perceptions of the accuracy of a predictive CDSS, willingness to act based on that predictive CDSS, as well as the factors that influence each of these and their relationship to each other. These concepts of interest were operationalized and explored using a semistructured interview guide (Table S1 in Multimedia Appendix 1).

### Study Design

Qualitative descriptive methodology guided our methods [18,19], which included directed deductive and inductive content analysis of interview data [20]. A semistructured interview guide (Table S1 in Multimedia Appendix 1) was developed by the research team and included questions guided by the human-computer trust framework as well as open-ended questions to elicit clinicians’ trust in predictive CDSSs generally and in the Communicating Narrative Concerns Entered by Registered Nurses (CONCERN) CDSS specifically. CONCERN is a predictive CDSS implemented at 2 hospitals within the Mass General Brigham health system that is currently under investigation for its ability to predict in-hospital deterioration (5R01NR016941-05). The system was implemented in July 2020 on 8 pilot units and in September 2020 on 16 additional study units. The study units included acute and intensive care units, excluding pediatric, neonatal, hospice, emergency, oncology, labor and delivery, behavioral or psychiatric, observational, perioperative, same-day surgery, and plastic surgery units.

**Figure 2:**
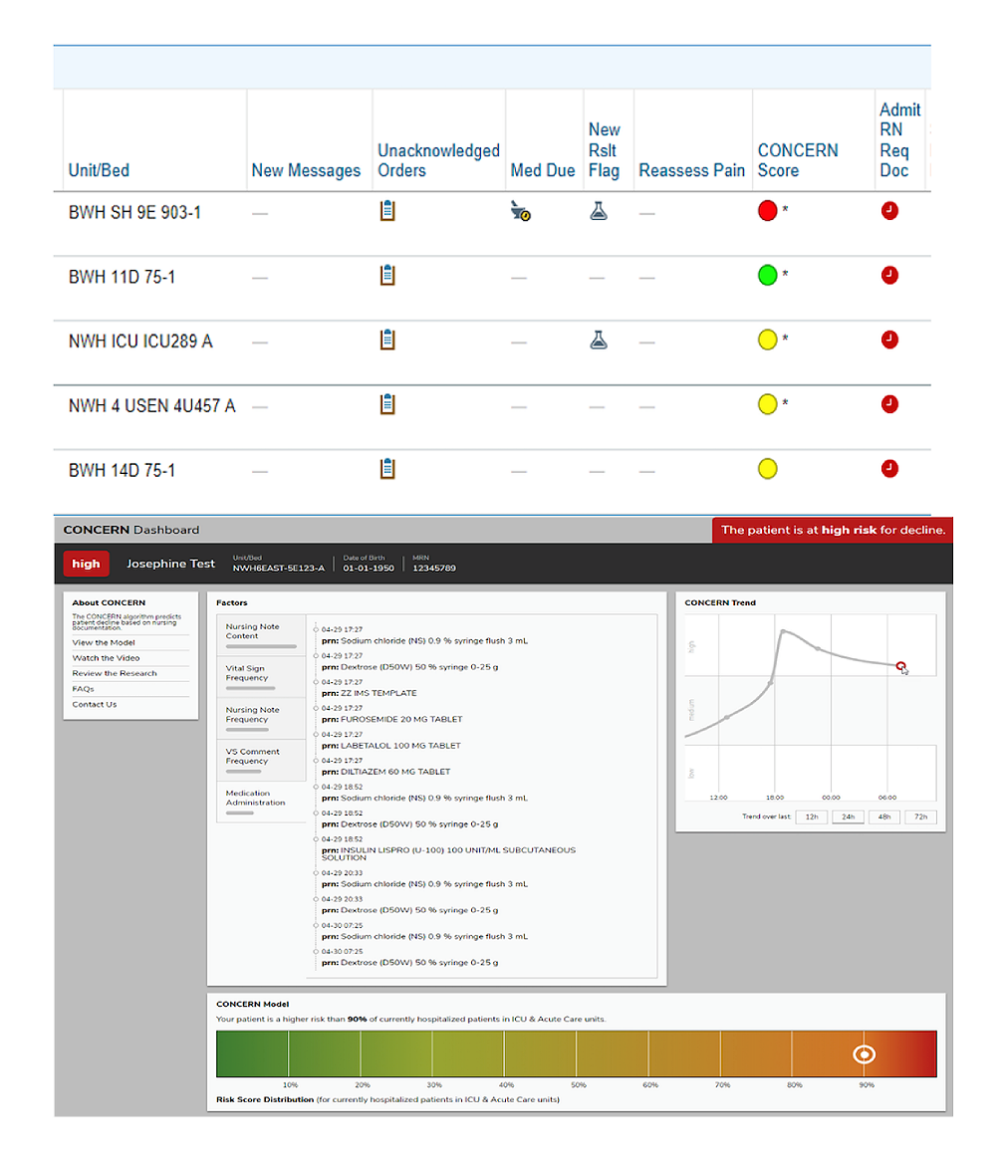
Communicating Narrative Concerns Entered by Registered Nurses (CONCERN) clinical decision support system.

CONCERN uses machine learning and natural language processing to model nursing documentation data for predicting patient risk of in-hospital deterioration. As such, it leverages evidence that nurses alter their documentation behavior or use selected language in their narrative notes when they are concerned about a patient’s changing clinical state [21-24]. As shown in Figure 2, CONCERN provides clinicians with a prediction in the form of a colored circle indicating patient risk of deterioration: green indicates low risk, yellow indicates increased risk, and red indicates high risk. By clicking on the color, clinicians open the CONCERN dashboard, which displays the 5 feature (ie, predictor) categories driving the prediction, the relative importance of each in informing that patient’s prediction, the patient-specific documentation contributing to each feature category, a trend line of the patient’s prediction across their admission, where the patient falls along the CONCERN distribution, and links to learn more about CONCERN’s development or provide feedback. The five overarching feature categories used in CONCERN predictions are (1) nursing note content, (2) vital sign frequency, (3) nursing note frequency, (4) vital sign comment frequency, and (5) medication administration.

### Participant Recruitment

CONCERN was added to the patient lists of all clinicians working in acute or intensive care units. However, predictions were only displayed for patients admitted to the study units (a random sample of 24 acute or intensive care units). Clinicians in these units received training on CONCERN, and then, 3 months after CONCERN was implemented in all study units (December 2020), clinicians using CONCERN were invited to enroll. Snowball sampling was also used (ie, participants were asked to advertise the study or refer their peers). Clinicians were not excluded if they had not elected to incorporate CONCERN into their regular practice as we did not want to bias our results toward only those who had a positive perception of CONCERN. Clinicians were characterized as either nurses or prescribing providers (physicians, PAs, and nurse practitioners). The participants received a US $50 gift card.

### Ethics Approval

The Institutional Review Boards at Columbia University Irving Medical Center (AAAR1389) and Brigham and Women’s Hospital (2019P001910) approved this study.

### Data Collection

A semistructured interview guide was used to iteratively direct each interview (Table S1 in Multimedia Appendix 1). Intentionally, we did not introduce the term *machine learning* at the outset of interviews to remain open to clinicians’ perceptions of working with predictive CDSSs in practice rather than to potentially bias their responses based on their perceptions of machine learning. Interviews were conducted remotely using Zoom (Zoom Video Communications) outside of clinicians’ work hours. Interview length ranged from 20 to 56 minutes (mean length 39, SD 9.5 minutes). Interviews were recorded and transcribed using a Health Insurance Portability and Accountability Act–compliant transcription software (NVivo Transcription; QSR International) and cleaned by the principal investigator (JMS).

We recruited participants until a data saturation table indicated that we had reached data adequacy (ie, no new information was being learned in subsequent interviews [25]) and we had a nearly equal number of nurses and prescribing providers so as to increase confidence in our comparison of findings between the 2 professional groups. Data saturation occurred at the 11th interview (Table S2 in Multimedia Appendix 1). We continued to recruit up to 17 interviews to balance our sample of professional groups.

### Data Analysis

Using both inductive and deductive directed content analysis, we created an initial codebook that defined our codes, established boundaries for their application, and included exemplar quotes [26] (Table S3 in Multimedia Appendix 1). The primary coder (JMS, a nursing informatics scientist) used this codebook to guide the coding of additional transcripts, revising the codebook as needed. A second coder (SRM, a nonclinical informatics graduate student) completed inductive coding of a purposive sample (half prescribing providers and half nurses) of 47% (8/17) of the transcripts. JMS and SRM met weekly to discuss their findings. Bimonthly debriefings with MG (a qualitative expert with no informatics background) served to achieve a consensus. A third coder (EL, a physician informaticist) completed deductive coding of a purposive sample (half prescribing providers and half nurses) of 35% (6/17) of the transcripts using the concepts of the human-computer trust framework. Intercoder reliability was calculated as Cohen *κ* coefficients to measure agreement between the coders performing deductive coding (JMS and EL). Consistent with the qualitative paradigm, the importance of codes was not determined by their absolute frequency [27]. Thus, we report both common and less common perceptions.

### Rigor of Data Collection and Analysis

We used multiple strategies for enhancing the trustworthiness of our findings as outlined by Guba [28]. To facilitate credibility (ie, to foster truth in our findings) [28], we used peer debriefing [29] and member checking (verifying emerging codes and categories in interviews with new participants) and assessed our final themes for structural corroboration (to confirm that the findings did not contradict each other). To enhance transferability (ie, the truthful representation of context and sample) [28], we report detailed demographic descriptions of our sample and site and sampled purposively to represent nurses and prescribing providers. To achieve dependability or consistency in the findings [28], we created the codebook and an audit trail documenting all data collection and analytic decisions made throughout the study. Finally, to foster confirmability (ie, reduce bias) [28], the coders practiced reflexivity to identify the researchers’ impact on the data. In addition, our interprofessional team of coders and researchers and our purposive sampling strategy that included enrollment of different clinician professions allowed us to triangulate our data; that is, to use multiple perspectives to increase our confidence in the study findings.

## Results

### Overview

We interviewed a total of 17 clinicians regarding their trust in predictive CDSSs generally and the CONCERN CDSS specifically. Overall, 53% (9/17) of the participants were prescribing providers (8/9, 89% physicians and 1/9, 11% PAs), and 47% (8/17) were nurses. Most clinicians (9/17, 53%) worked on general medicine units or rotations, they had an average of 5.43 years of experience in their current professional role, and an average age of 30.65 years. The participants reported working with the CONCERN CDSS for 1 to 6 months. Most clinicians (14/17, 82%) were recruited from 1 hospital (site A). Table 1 presents the aggregate participant demographics.

**Table 1:** Participant demographics (N=17).

Demographic variables					Values
**Clinician type, n (%)**					
	**Prescribing providers**			9 (53)	
		Physician	8 (47)		
		Physician assistant	1 (6)		
	**Nurses**			8 (47)	
		Registered nurse	7 (41)		
		Nurse educator	1 (6)		
**Current practice setting, n (%)**					
	Inpatient internal medicine			9 (53)	
	Cardiology, cardiac surgery, or vascular surgery			4 (24)	
	COVID-19 (previously internal medicine)			2 (12)	
	Surgery			1 (6)	
	Hospitalist			1 (6)	
Years in current profession, mean (SD; range)					5.43 (8.59; 0.5-35)
Years at Mass General Brigham, mean (SD; range)					6.12 (7.95; 0.5-32)
**Highest professional degree, n (%)**					
	Medical doctor			8 (47)	
	Bachelor of Science in Nursing			7 (41)	
	Master’s degree			2 (12)	
Age (years), mean (SD; range)					30.65 (8.66; 24-58)
**Race, n (%)**					
	Asian or Asian American			7 (41)	
	Biracial			1 (6)	
	White			8 (47)	
	Not reported			1 (6)	
**Ethnicity, n (%)**					
	Brazilian			1 (6)	
	Chinese			2 (12)	
	Eastern European			1 (6)	
	Hispanic			1 (6)	
	Korean or Korean American			2 (12)	
	Non-Hispanic			8 (47)	
	Not reported			2 (12)	
**Gender, n (%)**					
	Female			13 (76)	
	Male			4 (24)	
**Site, n (%)**					
	Site A			14 (82)	
	Site B			3 (18)	

### Deductive Coding Found Support for the Conceptual Framework

The 2 deductive coders achieved an overall Cohen κ of 0.81. We found support for the 2 concepts of the model: perceived technical competence (Cohen κ=0.77) and perceived understandability (Cohen κ=0.86).

#### Perceived Technical Competence

Clinicians described their trust as being influenced by their perceptions of the accuracy and correctness of CONCERN. For example, a physician said:

The more accurate it is, in my opinion...the more trust I have in the tool.

Physician 2

#### Perceived Understandability

Clinicians’ ability to understand CONCERN was also confirmed to be an important factor influencing trust. Clinicians described wanting to evaluate the factors contributing to CONCERN to determine whether they trusted the prediction (also referred to as the “score”):

The CONCERN score has changed, like, you know, they’re now a yellow or whatever, it might be a good point to be like, oh, what do we think is contributing to that or even reviewing like, because I think there’s a way to review, like what, what went into that. And just being like, do we trust this? Do we not?

Physician 4

### Inductive Coding

#### Overview

**Figure 3:**
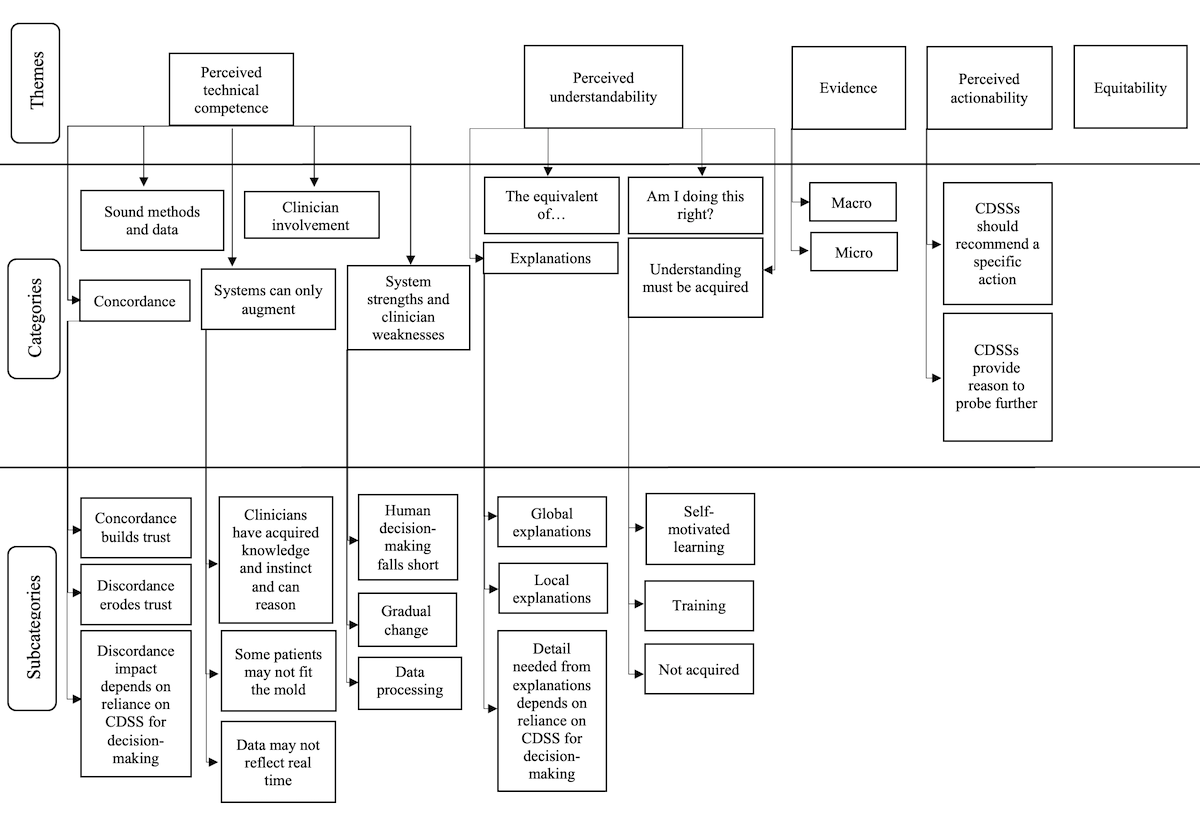
Themes, categories, and subcategories of factors influencing trust. CDSS: clinical decision support system.

The 2 concepts of cognition-based trust, *perceived technical competence* and *perceived understandability*, emerged as themes in the inductive coding. In addition, three new themes reflecting clinicians’ trust in predictive CDSSs were identified: (1) *evidence*, (2) *perceived actionability,* and (3) *equitability* (Figure 3). Emergent codes between sites A and B did not differ significantly (Table S2 in Multimedia Appendix 1).

#### Perceived Technical Competence

Regarding *perceived technical competence*, five categories characterized clinicians’ perceptions of the accuracy and correctness of CONCERN and predictive CDSSs: (1) *concordance*, (2) *sound methods and data*, (3) *clinician involvement*, (4) *systems can only augment*, and (5) *system strengths and clinician weaknesses.*

##### Concordance

Concordance between clinicians’ impression of the patient’s clinical status and CONCERN’s prediction emerged as an important factor influencing clinicians’ perceptions of the accuracy of CONCERN with (1) *concordance builds trust*, (2) *discordance erodes trust*, and (3) *discordance impact depends on reliance on CDSS for decision-making*.

##### Concordance Builds Trust

When CONCERN’s predictions aligned with clinicians’ impressions of the patient, their trust in the system was positively affected:

I felt good that it, very much aligned with how the patient was progressing, whether they were doing well or not doing so well.

registered nurse (RN) 1

Clinicians also hypothesized that, if there was concordance between their concern for a patient and the CONCERN prediction indicating high risk, they could use the prediction as evidence to support escalating care:

So, I guess in an instance, I could say that, like, oh, this patient’s CONCERN score is...red, like, this is just evidence that we need to do intervention.

RN 4

##### Discordance Erodes Trust

Conversely, clinicians expressed that a lack of concordance between CONCERN’s predictions and their assessments decreased their trust:

I think probably we all kind of take notice of it, but we don’t really talk about it because sometimes it doesn’t really correlate truly with how a patient is doing clinically.

PA 1

Similarly, a nurse stated:

That trust could be hindered, say, if I had a patient I was concerned about, and they were a green.

RN 4

##### Discordance Impact Depends on Reliance on CDSS for Decision-making

For some clinicians, discordance between the CONCERN prediction and their impression of the patient did not diminish their trust as they viewed CONCERN as just 1 data point that they considered. These clinicians described appreciating the prompt to pay attention to a patient and did not see any harm in an inaccurate prediction:

There have been moments where I’m like, “oh, I’ve been in there all day, why is it not red?” But again, it’s not frequent enough for me to say, “oh, this is garbage.” I, I still respect its input [laughs]...it’s something I look at at the start of my shift...as the day goes on, I am taking note if there is a change.

RN 3

The one time it was off, I think, was just there was a lot of documentation happening for other reasons that weren’t a good clinical deterioration. There was just a lot of other things going on with this patient that required frequent documentation. And so, it was like a yellow. But again, it was nice to just know, like, oh, I should actually kind of see what’s been going on.

Physician 1

##### Sound Methods and Data

When referring to the accuracy of CONCERN, many clinicians described their impression of the quality of data used in the model, data that could improve the model, and the modeling methods to varying levels of granularity. A few physicians expressed their endorsement of CONCERN’s methods because it leverages nursing documentation data:

My whole training has kind of just been like, trust your nurses, if they’re concerned, you’re concerned...And so I think any way that, like, further gives you insight into what is going on, on the nursing perspective, is helpful.

Physician 1

Others wanted to further scrutinize the rigor of model development:

I’d want to know a bit more about how it was developed, and so let’s say the data that CONCERN was trained on was exclusively ICU sepsis and organ failure, mortality, all-cause mortality, let’s say...then I would say this tool is only generalizable to the ICU setting, for example.

Physician 3

Many discussed the data quality, with some clinicians doubting that the frequency of nursing notes predicted deterioration:

[Nursing note] frequency, that only ever happens once a shift...I document my note at the end of my shift whether or not there is a significant event that happened.

RN 4

##### Clinician Involvement

Some participants wanted to know that clinicians had been involved in the development of the system or that clinicians would have the opportunity to provide feedback on system performance after its implementation. A nurse said:

I think just with anything, having someone who’s actually been there done that is way more, makes it, makes whatever you’re developing way more accurate, way more useful, way more diligent.

RN 5

##### Systems Can Only Augment

Clinicians identified several limitations to predictive CDSSs relative to clinicians and emphasized that CDSSs were just one of many sources of information that they considered when making clinical decisions. This category is illustrated through three subcategories: (1) *clinicians have acquired knowledge and instinct and can reason*, (2) *some patients may not fit the mold*, and (3) *data may not reflect real time*.

##### Clinicians Have Acquired Knowledge and Instinct and Can Reason

Clinicians described using their acquired expertise or gut instinct to mitigate patients’ risk of deterioration as well as their ability to put the objective data into context:

I feel like a lot of times we just kind of know when somebody is, like, not doing well, especially when we have the same patients often like day to day.

RN 1

You need those people to look at those numbers that are like patient’s tachycardic, heart transplant to say, “yeah, that’s abnormal, but it is normal.” And in a sense, you can’t really computerize that stuff. So that’s why a clinician’s judgment is so important...you need someone to be thinking, like, what do these numbers actually mean?

RN 4

##### Some Patients May Not Fit the Mold

Clinicians expressed skepticism of the system’s ability to account for unique or complex patient characteristics:

Because it’s so complicated, it’s quite, I mean, I hesitate to say unique, but there are a lot of a lot of factors in place. And it would be hard for a training data set to include enough patients who were similarly complex for it to have, let’s say, three hundred patients with infection in the right rib and the left shoulder and the left knee all at once. And for it [the system] to kind of know what to do at that point.

Physician 3

##### Data May Not Reflect Real Time

Clinicians frequently described scenarios in which data that the system uses would be missing. Examples included emergencies, rapid deterioration, new patients, or when clinicians are burdened by work or documentation:

I’m not saying that systems like this aren’t smart, but I just feel like so much of it depends on what’s going on in that moment. And a lot of times, you know, our documentation isn’t always like right up to date with what’s going on at the moment.

RN 7

##### System Strengths and Clinician Weaknesses

Clinicians also identified several reasons why predictive CDSSs might be more accurate than an expert clinician in predicting risk of in-hospital deterioration. This category is illustrated through three subcategories: (1) *human decision-making falls short*, (2) *gradual change*, and (3) *data processing*.

##### Human Decision-making Falls Short

Clinicians described their own and their colleagues’ limited abilities to make accurate predictions when they are tired, when they have limited experience (eg, either years in practice or with a particular patient population), when they are overly burdened, or when they are not “good” clinicians. For example, many clinicians mentioned that they would probably rely more on predictive CDSSs during the night shift, when they are assigned to care for more patients:

I’m like doing night coverage, so I don’t know the patients as well, so maybe I would, in that setting, be more reliant on a tool like that.

Physician 4

I’d say [CDSSs would be better than a clinician when] someone, a novice in their role. Like July [laughing about when new residents begin] or any new nurse or anything like that.

RN 3

##### Gradual Change

Clinicians described predictive CDSSs as better equipped to make predictions in situations where the change to the patient’s state is gradual rather than rapid:

Maybe the algorithm’s better at like kind of like nudging us to just like readdress some things that maybe are changing minutely day to day, so we may miss if we’re if we’re not, like, really aware of the trend.

Physician 4

##### Data Processing

Clinicians also recognized that there are large volumes of data to synthesize in the electronic health record (EHR) and that systems may be better equipped to process that volume of data, especially from clinical notes. Exemplar quotes are presented in Table S4 in Multimedia Appendix 1.

#### Perceived Understandability

*Perceived understandability* was characterized by four categories: (1) *explanations*, (2) *understanding must be acquired*, (3) *the equivalent of*... (physicians only), and (4) *Am I doing this right?* (nurses only).

##### Explanations

Much of the clinicians’ discussion of understanding CONCERN specifically and predictive CDSSs generally involved explanations of system logic and individual predictions. This category is presented in the form of three subcategories: (1) *global explanations*, (2) *local explanations*, and (3) *detail needed from explanations depends on reliance on CDSS for decision-making*.

##### Global Explanations

Clinicians wanted global explanations, meaning information on how the CONCERN model calculates predictions generally:

So my questions are like, well, what kinds of phrases and words and how often, you know, is the is the CONCERN tool looking back? Is it, are they looking at one note? Are they looking at three notes? And when you say vital sign frequency, what does that mean?

Physician 7

I think [to understand an algorithm like CONCERN] just more what it takes into account, whether it’s you know, their vital signs or their lab values, I don’t really know how it calculates, if they’re flagged as yellow or green.

RN 7

##### Local Explanations

Clinicians also wanted explanations for individual patient predictions provided at the point of care. A physician said they want to see “the vital signs or the whatever that is making the score change” (Physician 3). A nurse said they would look for “what piece of it is causing the algorithm to say that the person’s not stable” (RN 2).

##### Detail Needed From Explanations Depends on Reliance on CDSS for Decision-making

This third subcategory emerged from some clinicians stating that they did not need detailed explanations of CONCERN as it was just 1 component of their assessments:

The fact that it’s, it’s an extra data point that’s available to me there doesn’t make me so concerned about, well, you know, how does the machine learning work and when what goes into this? To me, I’m like, well, I understand what machine learning is and I understand that it helps me better inform some of my clinical decisions and maybe gives me like an extra reason to, to double check my, my own clinical assessment. So, in that sense, like I feel like it’s been a sufficient enough information for me.

Physician 8

##### Understanding Must Be Acquired

Clinicians described the various ways in which they came to understand CONCERN or did not understand it. This is described in three subcategories: (1) *self-motivated learning*, (2) *training*, and (3) *not acquired*.

##### Self-motivated Learning

In this subcategory, some clinicians described themselves as being self-motivated to learn. For example, they may have seen a poster or received an email about CONCERN that prompted them to read about it, look at the predictions more frequently, or investigate the dashboard. Exemplar quotes are presented in Table S4 in Multimedia Appendix 1.

##### Training

Some clinicians previously participated in CONCERN design focus groups, which they described as a helpful form of training, whereas others received formal training. Clinicians felt that formal training should be part of onboarding new staff. Exemplar quotes are presented in Table S4 in Multimedia Appendix 1.

##### Not Acquired

Conversely, some clinicians had a poor understanding of CONCERN, with a few who felt that they did not receive education expressing frustration about this. Exemplar quotes are presented in Table S4 in Multimedia Appendix 1.

Although all previous categories were informed by both prescribing providers and nurses, 2 categories were profession-specific: only physicians used analogies, and only nurses were concerned about how their documentation affected predictions.

##### The Equivalent of...

In this category, some physicians used analogies when describing their understanding of CONCERN:

I literally think of it the same as imaging, like I usually rely on radiology reports, because I’m not a radiologist, but I do like to look at the images myself because...you can sometimes have a different context for what you’re looking for that the radiologist doesn’t know.

Physician 4

##### Am I Doing This Right?

Knowing that their documentation would inform the CONCERN prediction, some nurses wanted to know that they were not missing something that would make the CONCERN prediction more accurate. Some said that they had or would change their documentation behavior in attempts to make the prediction reflect their impression of the patient:

I feel like I do try to put stuff in that’s like part of the CONCERN score, but it doesn’t always, like the CONCERN score doesn’t always reflect it, so then I’m like, I’m not sure I’m putting in the data correctly? Or like I’m just not putting it in the right comment boxes or like filling out my notes, you know, I don’t know if, like, I’m the one who’s not raising that level of concern because I’m just not putting, I’m not, like, doing the algorithm correctly where it would recognize it as a concern.

RN 6

Finally, new themes emerged from the data analysis that do not map onto the conceptual framework. These included (1) *evidence*, (2) *perceived actionability*, and (3) *equitability*.

#### Evidence

The *evidence* theme emerged from clinicians’ discussions of how evidence of CDSSs positively affecting patient care would increase their trust in the system’s predictions. In all, 2 categories emerged: *macro* and *micro*.

##### Macro

In *macro*, scientific evidence of the impact of a predictive CDSS on patient care was important for facilitating trust:

I think, really like a study showing that the score has been used and the evidence behind it...if it’s published and peer reviewed, I think I definitely, personally I’d be more more inclined to use it.

Physician 6

##### Micro

Clinicians also described the importance of anecdotal reports of positive impact:

I think anecdotally, like if others I know said, “hey, you know, I happened to catch this patient who was deteriorating and we were actually able to like, you know, get involved early and we were able to prevent this patient from either a rapid or like likely ICU transfer.” I think those things pull like a lot of weight.

Physician 1

#### Perceived Actionability

Some clinicians wanted a clear recommendation for an action to take to trust the predictive CDSS (which CONCERN does not provide). Others discussed how CDSSs provide reason for them to further examine a patient’s clinical status.

##### CDSSs Should Recommend a Specific Action

Some clinicians expressed a desire to know what to do with the patient’s risk score to trust it:

Understanding how the predicting part comes in, I think would give me more confidence...some sort of like if/then tool, so if the score is greater than this, then you should take this kind of action.

Physician 3

##### CDSSs Provide Reason to Probe Further

Clinicians also noted that a CDSS prediction indicating elevated risk had prompted or would prompt them to investigate further, either via EHR data review or discussion with another team member:

If you’re talking about trust, I feel like me looking at a red patient...it would boil down to, OK, this patient’s red, I want to look into their chart.

RN 4

If I saw a red or anything other than green, I’d click on that patient, look at their flowsheets and then if they were like tachycardic, two hours ago they were not, then I would go and actually visit the patient and check with the nursing team to see if they had any concerns.

Physician 3

#### Equitability

A clinician expressed the importance of the predictive model being equitable:

The one caveat [to machine learning] is it could, if it uses, you don’t know exactly what data it uses, and I would be interested in studies that explore whether that machine is systemically racist or classist or whatever...So, some sort of study to make sure it’s equitable to all populations is important.

Physician 6

## Discussion

### Principal Findings in the Context of What Is Known

Our qualitative descriptive investigation using the human-computer trust framework [17] produced broad and deep characterizations of nurses’ and prescribing providers’ trust—and distrust—in predictive CDSSs. We confirmed that perceived understandability and perceived technical competence influence clinicians’ trust in predictive CDSSs as well as identified additional factors: evidence, perceived actionability, and equitability. Furthermore, we found profession-specific factors characterizing the relationship between understandability and trust.

Although we focused on cognition-based trust, our findings have implications for reconceptualizing the human-computer trust framework. In each interview, the clinicians were asked what would increase or decrease their trust in CONCERN. In all, 3 concepts of the framework (perceived reliability, faith, and personal attachment) were not identified by the participants. However, these concepts might be more dependent on sustained system use, which not all of the participants had. Other works conceptualize trust as being influenced by an individual’s propensity to be trusting [11]. Although this concept also did not emerge definitively in our study, it is possible that the clinicians who described being self-motivated in their learning about CONCERN were indirectly demonstrating a propensity to be trusting.

Much has been written about the importance and perceived trade-off between accuracy and understandability in machine learning–based CDSSs [30,31]. Our investigation found that both are important and provided context for clinicians’ desires for each in the case of predictive CDSSs. As CONCERN was implemented during the COVID-19 pandemic, there were limited opportunities for in-person education, and there were increased demands on the clinical staff. This may have contributed to clinicians having a poor understanding of CONCERN and its global and local explanations on the dashboard. In fact, when some clinicians were asked how they would determine that the CONCERN tool was accurate, they answered by expressing a desire to understand it more thoroughly—indicating a primacy of understandability over accuracy, as others have found [2]. This preference may differ for machine learning–based diagnostic CDSSs, as hypothesized by Diprose et al [6].

However, delivering an accurate and *desirable* explanation of machine learning logic remains a challenge. When describing their desire for local explanations, many clinicians indicated an orientation toward rule-based causal logic. They wanted to know the one feature or value that made the patient’s prediction yellow or red. In the case of many predictive CDSSs, such simplifications are not possible, and an interpretation of causation would not be accurate. When our team iterates on the CONCERN design, we will look to explanation design frameworks such as that outlined by Barda et al [32] to optimize the impact of explanations on understandability. However, long-term strategies aimed at increasing the education that clinicians receive on machine learning are also likely needed, as others have also reported from their investigations [7,33,34].

We found that some of the factors influencing clinicians’ perceptions of system accuracy (ie, perceived technical competence) differed from findings in previous research. Tonekaboni et al [2] reported that clinicians would like to see a certainty score presented with the CDSS prediction; however, no clinician requested this or any type of accuracy metric in our interviews. When prompted, they said that an accuracy metric would be helpful, but differences may be attributable to context. Tonekaboni et al [2] interviewed clinicians referring to simulated rather than implemented predictive CDSSs. We found that clinicians primarily judged the accuracy of CONCERN against their own impressions of their patients’ risk of deterioration, which may be what clinicians do in real clinical care. In fact, this was suggested by clinicians in the study by Tonekaboni et al [2].

Importantly, many of the categories that inductively emerged in this study align with others’ findings. For example, Sandhu et al [7] reported that “even when physicians did not trust a model output, they still reported paying closer attention to a patient’s clinical progression,” aligning with our category *CDSSs provide reason to probe further.* Elish [35] also found that evidence was important to clinicians, particularly “anecdotal evidence and discussions of specific cases and patient outcomes,” aligning with our category *micro evidence.* In addition, many have highlighted the importance of engaging clinical end users throughout development and implementation [7,35-38]. However, most predictive CDSS studies do not report involving clinicians in development, indicating that this is an area for future work [39].

Others have warned about an overreliance on inaccurate machine learning–based CDSS predictions or classifications [30,40]. In fact, Jacobs et al [15] found that clinicians trusted incorrect recommendations. Similarly, Cabitza et al [40] argued that clinical users of machine learning–based CDSSs using EHR data need to be aware that data “quality is far from optimal” and warned clinicians about losing awareness of important clinical factors not present in the EHR. However, the clinicians in our study did not show a propensity to overrely on the CONCERN predictions and indicated that they recognized predictive CDSSs’ shortcomings.

Only nurses in our study wanted to understand how to document “correctly” for the CONCERN score, with some indicating that they would change or had already changed their documentation behavior to make the CONCERN score more accurate (in their estimation). This has implications for model performance as well as documentation burden, as CONCERN was intentionally designed to work without adding documentation to clinicians’ workload. It also reflects a paradigm shift. Nurses are accustomed to rule-based scoring systems such as Morse Fall Risk [41] in which they enter clear assessment points to directly calculate a risk score, whereas CONCERN uses machine learning to model existing documentation without soliciting direct input from clinicians. As predictive CDSSs such as CONCERN do not involve that direct connection, nurses may require direct connections to patient outcomes or more thorough and detailed local explanations to trust predictions. Finally, only physicians used analogies to describe their understanding of CONCERN. This may be reflective of the contention by Lee [42] that humans tend to anthropomorphize goal-directed intelligent systems and may be unique to physicians in this study because CONCERN leverages nursing rather than physician documentation.

### Limitations

This study has several limitations. Our use of the human-computer trust framework [17] may have biased clinicians toward certain conceptualizations. For example, we prompted clinicians to compare predictive CDSSs with expert clinicians as guided by the operationalization of perceived technical competence by Madsen and Gregor [17]. Without this prompt, clinicians may not have compared their abilities with the abilities of predictive CDSSs. Our specific questions about CONCERN (and about predictive CDSSs generally) as well as the heterogeneity in exposure to CONCERN limit our ability to know which findings are unique to CONCERN. Future research with other predictive CDSSs should control system exposure to further characterize the phenomenon of clinician trust. As with any qualitative research, our findings may not be transferable to other settings and populations. For example, perceptions may be different among older clinicians whose training and residency may not have involved EHRs and CDSSs. We also did not successfully recruit any clinicians who worked in intensive care; therefore, our findings may not be transferable to clinicians using predictive CDSSs in intensive care settings. There are also limitations inherent to remote interviews over video. We had limited ability to read nonverbal language, and 6% (1/17) of the participants opted not to turn on their camera. However, field notes were taken during the interviews, capturing tone of voice and nonverbal language. Finally, social desirability may have affected responses as the participants knew that the interviewer was with the CONCERN team, which may have led them to self-censor negative perceptions of CONCERN.

### Implications for Research, Practice, and Policy

The findings of this investigation elucidate future areas of inquiry. First, it will be important to explore the differences in requirements for trust between differing versions of predictive CDSSs. For example, CONCERN does not recommend a discrete action, whereas other systems pair predictions with a recommended action. We found that clinicians’ preferences for a recommended action varied and influenced trust. Furthermore, the extent to which clinicians rely on the predictive CDSS was shown to influence both the impact of discordant predictions and the detail needed from explanations. This may indicate that predictive CDSSs that are prescriptive or essential to the workflow will require more concordance or explanation detail than those that are informative, such as early warning systems.

It will also be important to evaluate the reception of CONCERN’s global and local explanations over sustained use and with reinforced education. It is clear from these findings that clinicians are oriented toward rule-based logic and this should be accounted for in explainable artificial intelligence research. Future research should also investigate whether nurses using CONCERN in fact change their documentation and, if so, whether those changes result in increased documentation burden or variation in predictive model performance. Finally, future work should be dedicated to investigating clinician personal attributes that may contribute to the variation in factors influencing trust.

It may be advantageous for hospital administrators to implement policies for development and implementation of predictive CDSSs aimed at increasing trust and adoption. Our findings suggest that involving clinicians in model development, allowing them to provide feedback after implementation, designing user-centered explanations for predictive CDSSs, and educating clinicians on machine learning may be effective policies for increasing trust.

### Conclusions

Clinician trust in predictive CDSSs is critical for increased adoption of data-driven patient care. Our investigation of the phenomenon of clinician trust in predictive CDSSs for in-hospital deterioration produced needed knowledge on the factors that influence clinician trust. We found that perceptions of trust were largely the same between nurses and prescribing providers. Future work should investigate the relationship between perceived actionability and trust, research explanations that enhance understandability, and explore policies aimed at facilitating trust.

## Appendix

Multimedia Appendix 1 Research materials and extended results.

## Abbreviations

CDSS: clinical decision support system
CONCERN: Communicating Narrative Concerns Entered by Registered Nurses
EHR: electronic health record
PA: physician assistant
RN: registered nurse
