# Supplementary material for: Factors Influencing Clinician Trust in Predictive Clinical Decision Support Systems for In-Hospital Deterioration: Qualitative Descriptive Study
Source: JMIR Hum Factors. 2022 May 12;9(2):e33960. doi: 10.2196/33960 (PMC9136656; doi:10.2196/33960)
Supplement: Multimedia Appendix 1 [file humanfactors_v9i2e33960_app1.docx]

Supplementary Tables

Table S1. Semi-structured interview guide

| Definitions | I’m going to be asking you some questions about your trust in clinical decision support that uses predictive algorithms and the CONCERN tool which is a decision support tool on your unit that uses a predictive algorithm to predict patient deterioration based on nursing documentation.  Using a predictive algorithm means using computations to map data on patient factors to target outcomes, such as death or cardiac arrest, rather than relying on a human expert.  I’ll be defining your trust in these systems as, “the extent to which [you are] confident in, and willing to act on the basis of, the recommendations, actions, and decisions of these systems” |
| --- | --- |
| Broad, opening questions | - When I say the word clinical decision support (CDS) what do you think of? - Tell me about your experiences with CDS tools in practice. - Tell me about your experiences with the CONCERN tool in practice. |
| Trust | - Tell me about a time when you trusted CONCERN’s predictions.   1. What made you trust CONCERN’s prediction in this case? - What else makes you trust CONCERN’s predictions? - Tell me about a time when you didn’t trust CONCERN’s predictions   1. What made you distrust CONCERN’s prediction in this case? - What else makes you distrust CONCERN’s predictions? - What do you think would increase your trust in CONCERN? - What do you think would decrease your trust in CONCERN? |
| Perceived Understandability | - What information do you need to understand a predictive algorithm such as the CONCERN algorithm? - Describe to me your understanding of how the CONCERN algorithm works.   1. What information does it use? - What has helped you to understand the CONCERN tool?   1. What was helpful about it? - What has hindered you from understanding the CONCERN tool?   1. What was hindering about it? - How does your understanding of how the CONCERN algorithm works influence your trust in its predictions? |
| Perceived Technical Competence | - Is the information that CONCERN uses good information? - When is an expert clinician better than a CDS tool at predicting patients at risk for deterioration?   1. Conversely, when is a CDS tool better than an expert clinician? - Let’s say we are creating a predictive algorithm for a CDS tool that is most accurate in predicting patient deterioration, what information does it use? - How does your impression of the CONCERN tool’s accuracy and correctness influence your trust in its predictions? - Our research team is able to set the CONCERN algorithm so that a certain percentage of patients are in each category – green, yellow, red. We’ve set it, based on clinician feedback. Given this fact, that we can engineer these categories, do you think the CONCERN algorithm is better at identifying patients in one of the categories compared to the others?   1. Which category? Tell my why. |
| General | - Tell me about a time when you felt the CONCERN prediction aligned well with your impression of the patient. - Tell me about a time when you felt the CONCERN prediction did not align with your impression of the patient, if you’ve experienced this. |

Table S2. Data saturation table

|  | 1 | 2 | 3 | 4 | 5 | 6 | 7 | 8 | 9 | 10 | 11 | 12 | 13 | 14 | 15 | 16 | 17 |
| --- | --- | --- | --- | --- | --- | --- | --- | --- | --- | --- | --- | --- | --- | --- | --- | --- | --- |
| Theme  Category  Sub-category | MD | RN | MD | MD | MD | MD | MD | PA^a^ | MD^a^ | RN^a^ | MD | RN | RN | RN | RN | RN | RN |
| Perceived Technical Competence |  |  |  |  |  |  |  |  |  |  |  |  |  |  |  |  |  |
| Concordance |  |  |  |  |  |  |  |  |  |  |  |  |  |  |  |  |  |
| Concordance Builds Trust | X | * | * |  | * |  |  | * | * |  |  |  | * | * | * | * | * |
| Discordance Erodes Trust |  |  |  |  |  |  |  | X | * | * | * |  | * |  | * | * |  |
| Discordance Impact Depends on Reliance on CDSS for Decision-making | X | * |  |  |  |  |  |  |  |  |  | * |  |  | * | * | * |
| Sound Methods and Data | X | * | * | * | * | * | * | * | * | * | * | * | * | * | * | * | * |
| Clinician Involvement | X |  |  |  | * |  |  |  | * |  |  |  | * | * | * | * | * |
| Systems Can Only Augment |  |  |  |  |  |  |  |  |  |  |  |  |  |  |  |  |  |
| Clinicians Have Acquired Knowledge, Instinct, and Can Reason | X | * | * | * |  |  | * | * |  | * |  | * | * | * | * |  | * |
| Some Patients May Not Fit the Mold |  | X | * | * | * | * |  |  | * |  |  | * | * |  |  |  |  |
| Data May Not Reflect Real Time | X | * | * |  | * |  | * |  |  |  | * | * | * | * | * | * |  |
| Systems strengths, clinician weaknesses |  |  |  |  |  |  |  |  |  |  |  |  |  |  |  |  |  |
| Human Decision-making Falls Short | X | * | * | * | * | * | * |  | * | * | * | * | * | * | * |  | * |
| Gradual Change | X |  |  |  | * |  |  |  |  |  |  |  |  | * | * |  |  |
| Data Processing |  |  |  | X |  | * |  |  | * | * | * |  |  |  |  |  |  |
| Perceived understandability |  |  |  |  |  |  |  |  |  |  |  |  |  |  |  |  |  |
| Explanations |  |  |  |  |  |  |  |  |  |  |  |  |  |  |  |  |  |
| Global Explanations | X |  |  | * | * | * | * | * | * | * |  | * | * | * | * | * | * |
| Local Explanations |  |  |  | X | * | * |  |  | * | * |  | * | * | * | * | * | * |
| Detail Needed in Explanations Depends on Reliance on CDSS for Decision-making |  |  |  |  |  |  |  |  |  |  | X |  |  | * |  |  | * |
| Understanding must be acquired |  |  |  |  |  |  |  |  |  |  |  |  |  |  |  |  |  |
| Self-motivated learning |  |  |  |  |  |  | X |  | * |  | * |  | * | * |  |  |  |
| Training | X |  | * |  | * |  | * | * | * | * |  | * | * | * | * | * | * |
| Not Acquired |  |  |  | X | * | * | * | * |  |  | * | * | * | * | * | * | * |
| The equivalent of… | X |  | * | * | * | * |  |  |  |  |  |  |  |  |  |  |  |
| Am I doing this right? |  | X |  |  |  |  |  |  |  |  |  | * | * | * | * |  | * |
| Evidence |  |  |  |  |  |  |  |  |  |  |  |  |  |  |  |  |  |
| Macro |  |  | X |  | * | * | * | * | * | * |  | * |  | * |  | * |  |
| Micro | X |  |  |  | * |  | * | * | * | * | * |  |  | * |  | * |  |
| Actionability |  |  |  |  |  |  |  |  |  |  |  |  |  |  |  |  |  |
| CDSSs Should Recommend a Specific Action |  |  |  | X |  | * | * |  | * |  |  | * | * |  |  |  |  |
| CDSSs Provide Reason to Probe Further | X | * |  | * |  | * | * |  |  | * | * | * | * |  |  | * | * |
| Equitability |  |  |  |  |  |  | X |  |  |  |  |  |  |  |  |  |  |

*Note.* The interviews are presented chronologically from left to right. X denotes the first interview in which a code/category was identified and * denotes subsequent use. ^a^Clinician from Site B as opposed to Site A.

Table S3. Sample of codebook

| Code | Brief Definition | Full Definition | When to Use | When not to Use | Example |
| --- | --- | --- | --- | --- | --- |
| CDSSs should recommend a specific action | Direct action recommended to clinicians by CDSS | CDSSs may provide a direct recommendation of what to do in terms of patient care or management | Apply to discussion or questions seeking to know what to do with CONCERN CDSS or hypothetical CDSS and the desire to have a clear directive from the CDSSs | Do not apply to discussion of the CDSSs making the clinician think or go see the patient and investigate without a clear directive | "I think in general, if I had to kind of make a conclusion about how I use these tools, is I generally trust tools that lead to discrete endpoint, like it really influences the way I manage patients." |
| CDSSs provide reason to probe further | CDSS makes clinicians think or investigate about the patient | CDSSs can provide a score or indicator that catches the clinician’s attention or changes their awareness of a patient, potentially prompting them to investigate to understand the meaning behind the prediction/indicator | Apply to comments about CONCERN or a hypothetical CDSS making clinicians think more about their patients, discuss with colleagues, go see the patient, or read through their chart. | Do not apply to discussion of the CDS tool providing a direct recommended action to clinicians | "Algorithms that will assist oftentimes fatigued or suboptimal clinicians and guiding towards either clinical questions to help to, to probe the mind a little bit more"  "It's more caught my attention when everything looks fine from a vitals standpoint, but there was someone with a yellow or a red. And so it made me kind of either click into the chart and see kind of what was being documented to get more context or if it wasn't clear and evident, I would obviously just call the nurse right away and just get more context." |

*Note.*  Modeled after MacQueen et al. (1998)

Table S4. Exemplar quotes

| Data processing | “Knowing that it contains qualitative information that is really difficult for me to process, is helpful in knowing, OK, this is a way of helping to synthesize that qualitative information, which is helpful because we can't, because a lot of the things that we rely on are quantitative, whether it's lab results, vital signs, whatever.” (Physician 8)  “The challenge is, I, as an intern am not continuously looking through the nursing notes, because there's so many notes for each patient.” (Physician 5)  “I think of essentially a set of algorithms that are able to look at trends and data which perhaps are not as apparent to a human observer for whatever reason, maybe there's just a lot of data or maybe the human is tired, whereas machines don't get tired, and they can process significantly larger amounts of data.” (Physician 3) |
| --- | --- |
|  |  |
| Self-motivated learning | “But I wonder if the nurses even know to click into the score. And the only re- I feel like the only reason why I clicked into it was because I felt like I wanted to be a part of this project and I wanted to know more about the project in and of itself.” (RN 4)  “It [education on CONCERN] felt pretty self-motivated because it just showed up one day and we all said, ‘what the heck is this?’…And then I think after getting a couple of emails, read the flyer, said something along the lines of, ‘yeah! this might be a good idea, umm and the worst I can do is ignore this, like all the other columns that I have that I ignore on a regular basis’, popped it back in… I'm hanging out on pod and…there is the like gigantic CONCERN poster that's right beside the um the nutrition station, which is very strategically placed, as I'm waiting for whatever, like the ice to like fill up, it gives me some time to peruse and enough that I'm like, ‘OK, actually this is something that I could use in my clinical practice.’ I don't know if everybody is like that.” (Physician 8) |
| Training | “I don't know if they know what it is or if they utilize it because, you know, we tried to do a lot of teaching when this first kind of rolled out. But as we've gotten, we have kind of a new team every month, so I don't know if the other providers are utilizing it.” (PA 1)  “I think what helped was having done the focus group kind of before, I think it was kind of explained to me like what was going into it.” (Physician 1)  “I guess like actually like having somebody go around and explain it… like, you know, the nurse educator, like how she comes around and she updates us on, like, the monitors and whatever, the educator. So she, maybe having someone come by and just be like, ‘hey, did you guys know there's this CONCERN score?’” (RN 8) |
| Not acquired | “I would say that I wasn't given much training as to how I should be using it. And so I I guess I keep an eye out for it and I take some time to look through it, understand what it means. But I would say that I don't yet like fully use or think that I can use it to its full potential.” (Physician 3)  “Well, I don't know if like, again, I feel like I still don't have a clear idea, like what exactly is feeding into it, so that's still my main question.” (Physician 4)  “Yeah, I'm still not sure I understand every, like I know we got like in an email, like different things that could cause the CONCERN score to increase… But like it wasn't like a list of phrases that you can use that would like kind of give the system an idea saying, oh, this is like this will like start, I don't know what you would call it, increasing the CONCERN score?” (RN 6) |
